# Chemoradiotherapy with 3-weekly CDDP 80 mg/m^2^ for head and neck squamous cell carcinoma: 5-year survival data from a phase 2 study

**DOI:** 10.3389/fsurg.2022.1035349

**Published:** 2022-12-16

**Authors:** Kohei Otaki, Takeshi Takahashi, Ryoko Tanaka, Kohei Saijo, Jo Omata, Yusuke Yokoyama, Ryusuke Shodo, Yushi Ueki, Keisuke Yamazaki, Hisayuki Ota, Takafumi Togashi, Nao Takahashi, Ryuichi Okabe, Hiroshi Matsuyama, Arata Horii

**Affiliations:** ^1^ Department of Otolaryngology Head and Neck Surgery, Niigata University Graduate School of Medical and Dental Sciences, Niigata, Japan; ^2^ Department of Head and Neck Surgery, Niigata Cancer Center Hospital, Niigata, Japan; ^3^ Department of Otorhinolaryngology, Nagaoka Red Cross Hospital, Niigata, Japan; ^4^ Department of Otorhinolaryngology, Niigata City General Hospital, Niigata, Japan

**Keywords:** head and neck cancer, squamous cell carcinoma, chemoradiotherapy, 3-weekly CDDP 80 mg/m 2, 5-year survival data, late adverse events

## Abstract

**Objective:**

The global standard for chemoradiation therapy (CCRT) for head and neck squamous cell carcinoma is cisplatin 100 mg/m^2^ administered once every three weeks, although cisplatin 80 mg/m^2^ is also widely used as an alternative treatment to reduce adverse events in Japan. We aimed to assess the long-term survival outcomes and late adverse events associated with CCRT with a 3-weekly cisplatin dose of 80 mg/m^2^.

**Methods:**

A phase 2 study on CCRT with a 3-weekly cisplatin dose of 80 mg/m^2^ was performed in 47 patients between April 2015 and December 2016 at four centers in Japan. Survival outcomes and late adverse events at 5 years after this phase 2 trial were investigated.

**Results:**

The median follow-up period was 61 months. The 5-year progression-free survival/overall survival of all 47 patients was 66.0%/76.6%, while that of patients with stage III, IV disease (UICC) was 65.6%/71.9%. Seventeen patients (36%) experienced dysphagia as a late adverse event. Univariate and multivariate analyses revealed a significant association between acute mucositis/low body mass index (BMI) during CCRT and late dysphagia.

**Conclusion:**

The survival outcomes of CCRT with a 3-weekly cisplatin dose of 80 mg/m^2^ may be comparable to the previously reported dose of 100 mg/m^2^. Acute mucositis and low BMI at CCRT were risk factors for late dysphagia, indicating the importance of managing these conditions during CCRT to prevent late adverse events. Caution and care for acute mucositis and swallowing training in patients with low BMI may be important for preventing late-stage dysphagia.

## Introduction

High-dose cisplatin (CDDP) is the most common concurrent chemoradiotherapy (CCRT) regimen for advanced head and neck squamous cell carcinoma (HNSCC), and the National Comprehensive Cancer Network guidelines recommend a CDDP dose of 100 mg/m^2^ every three weeks (1). In contrast, in Japan, since the CCRT completion rate at the 100 mg/m^2^ dose was as low as 59.8%–85% due to adverse events (2, 3), a reduced dose of 80 mg/m^2^ is widely used. Moreover, a CDDP dose of 100 mg/m^2^ for HNSCC is not covered by insurance in Japan. From 2015 to 2016, we conducted a multicenter phase 1/2 study to validate CCRT with a CDDP dose of 80 mg/m^2^, and the findings revealed a high CCRT completion rate (93.6%) and a favorable response rate (93.6%) at the first assessment of tumor response (4). Although these initial results were favorable, an assessment of long-term survival is essential. Moreover, late adverse events, particularly late dysphagia, which was evident in the RTOG91–11 trial, require careful evaluation (5). Among the long-term outcomes and late adverse events of CCRT with a CDDP dose of 100 mg/m^2^, the 5-year overall survival (OS), 5-year progression-free survival (PFS), and incidence of dysphagia as an adverse event were 44%–76.8% (6–10), 28%–49.8% (6, 7), and 15%–24% (5, 9), respectively. However, these effects have not been examined at 80 mg/m^2^. If the long-term treatment outcomes at 80 mg/m^2^ are equivalent to those of at 100 mg/m^2^, 80 mg/m^2^ may be considered a promising treatment option, since a reduced dose of CDDP would reduce sequelae such as neurotoxicity, ototoxicity, and renal dysfunction that sometimes interfere with post-CCRT chemotherapy for recurrent/metastatic diseases. Moreover, late adverse events and their predictors provide important information regarding CCRT with a CDDP dose of 80 mg/m^2^. Therefore, we investigated the long-term outcomes and adverse events of CCRT for HNSCC five years after a previous clinical trial (4). In this study, the 5-year PFS, 5-year OS, late adverse events, and their predictors following CCRT with a 3-weekly CDDP dose of 80 mg/m^2^ were investigated.

## Materials and methods

### Study design

This study was approved by the institutional review board of Niigata University Medical and Dental Hospital (approval number: 2020–0384). Patients were prospectively enrolled in a previous phase 2 trial (4), and clinical data were retrospectively collected 5 years after enrollment.

### Patients

A previous phase 2 trial for CCRT with 3-weekly CDDP at a dose of 80 mg/m^2^ enrolled 47 patients between April 2015 and December 2016 at four centers in Japan (4). They were judged to be medically suitable for definitive chemoradiotherapy with an Eastern Cooperative Oncology Group performance status (PS) of 0 or 1 and normal hematopoietic, hepatic, and renal function without distant metastasis. The patient characteristics and treatment information for CCRT are shown in Table 1. The patients' age range was 40–74 years (median, 64 years). The study included 39 men and eight women, whose body mass index (BMI) ranged from 17.9–34.1 kg/m^2^ (median, 22 kg/m^2^). The PS was 0 in 45 patients and 1 in two patients. The primary sites were the nasopharynx, p16-positive oropharynx, p16-negative oropharynx, p16-unknown oropharynx, hypopharynx, and larynx in nine, 13, two, two, 17, and four patients, respectively. Stages according to the UICC 7th edition criteria, which were used when the phase 2 trial was being conducted, were II, III, IV-A, and IV-B in 5, 16, 22, and four patients, respectively. The stages reclassified using the UICC 8th edition criteria were I, II, III, IV-A, and IV-B in 6, 9, 16, 13, and three patients, respectively. CCRT completion rate, defined as the rate of completion of radiotherapy (RT) and administration of 200 mg/m^2^ or more of CDDP, was 91.5% (43 of 47 patients). Among the adverse events assessed using Common Terminology Criteria for Adverse Events (CTCAE) v5.0, grade 3 or higher mucositis was observed in 25 out of 47 patients (53%) in the acute stage of the phase 2 trial (4). Treatment response evaluated by the Response Evaluation Criteria in Solid Tumors (RECIST) criteria version 1.1 was complete response (CR)/partial response (PR)/progressive disease (PD) in 38 (80.1%)/6 (12.8)/3 (6.4%) patients according to endoscopic view, computed tomography, and magnetic resonance imaging at 4–6 weeks after and/or positron emission tomography-computed tomography at 11–12 weeks after the end of CCRT (4).

**Table 1:** Characteristics of the patients in the previous phase 2 trial of CCRT with 3-weekly CDDP at a dose of 80 mg/m^2^.

Characteristics	Variables (*n* = 47)	
Age, years		
Median (range)	64 (40–74)	
Sex, *n*		
Men	39	
Women	8	
Body mass index, kg/m^2^		
Median (range)	22 (17.9–34.1)	
ECOG performance status, *n*		
0	45	
1	2	
Primary site, *n*		
Nasopharynx	9	
Oropharynx (p16+)	13	
Oropharynx (p16-)	2	
Oropharynx (p16 unknown)	2	
Hypopharynx	17	
Larynx	4	
T-category, *n*	UICC7th	UICC8th
1	8	8
2	22	22
3	11	11
4	6	6
N-category, *n*		
0	9	9
1	10	15
2	26	21
3	2	2
Stage classification, *n*		
I	0	6
II	5	9
III	16	16
IV-A	22	13
IV-B	4	3
Complete CCRT, *n* (%)	43 (91.5)	
Acute adverse events		
Acute mucositis ≥ Gr3, *n* (%)	25 (53.2)	
Treatment Response		
Complete response, *n* (%)	38 (80.1)	
Partial response, *n* (%)	6 (12.8)	
Progressive disease, *n* (%)	3 (6.4)	

CCRT, concurrent chemoradiotherapy; ECOG, eastern cooperative oncology group; UICC, union for international cancer control.

### Procedures

Using 4–10-MV x-rays, radiotherapy (RT) was delivered in a standard fractionation of 40–50 Gy (2 Gy/fraction, once a day) to level II through IV lymph nodes on both sides, and 66–70 Gy was delivered to the primary lesion and metastatic nodes. Intensity-modulated radiation therapy was permitted for nine patients with nasopharyngeal carcinoma. Concomitant chemotherapy was 3-weekly CDDP at a dose of 80 mg/m^2^. Details of the infusions and radiotherapy quality assurance have been outlined previously (4).

The patients were assessed at regular intervals after CCRT completion. Follow-up examinations were conducted every 1–2 months for 2 years and every 3–4 months thereafter. More frequent visits were recommended at the discretion of the individual clinicians.

### Outcome measurements

The primary endpoint was PFS, defined as the time from the initiation of CCRT until disease progression or death. The secondary endpoint was OS, defined as the time from the initiation of CCRT until death. Late adverse events, including dysphagia, dysphonia, hypothyroidism, and aspiration pneumonia, were assessed at 5 years after the completion of CCRT or just before recurrence or death. The functional oral intake scale (FOIS) (11) and performance status scale for head and neck cancer patients (PSS-HN) (12) were used to assess dysphagia and dysphonia, respectively. The FOIS scores are shown in Table 2. In this study, as in previous reports of swallowing evaluation after CCRT (13), FOIS level V or less was defined as dysphagia: patients with level V require rice porridge, whereas those with level VI can eat rice without special preparation. In Japan, rice porridge is a major meal for patients with dysphagia. Post-irradiation hypothyroidism was defined by the need for levothyroxine prescription under regular monitoring of thyroid-stimulating hormone levels. History of hospitalization for aspiration pneumonia was also assessed.

**Table 2:** Functional oral intake scale (FOIS) (11).

Level I	Nothing by mouth.
Level II	Tube dependent with minimal attempts of food or liquid.
Level III	Tube dependent with consistent oral intake of food or liquid.
Level IV	Total oral diet of a single consistency.
Level V	Total oral diet with multiple consistencies,
	but requiring special preparation or compensations.
Level VI	Total oral diet with multiple consistencies
	without special preparation, but with specific food limitations.
Level VII	Total oral diet with no restrictions.

To identify the risk factors for dysphagia (FOIS level V or lower), univariate and multivariate analyses were performed for age, sex, BMI, primary site, T-category, stage classification using the UICC 8th version, and acute mucositis.

### Statistical analysis

Survival curves were generated using the Kaplan–Meier method. Univariate and multivariate survival analyses were performed using the Cox proportional hazards model. Multivariate logistic regression was used to identify the risk factors for dysphagia. All statistical analyses were performed using EZR (Easy R; the R Foundation), which is now being distributed on the following website: http://www.jichi.ac.jp/saitama-sct/. Statistical significance was set at *P* < 0.05.

## Results

### Survival outcomes

**Figure 1:**
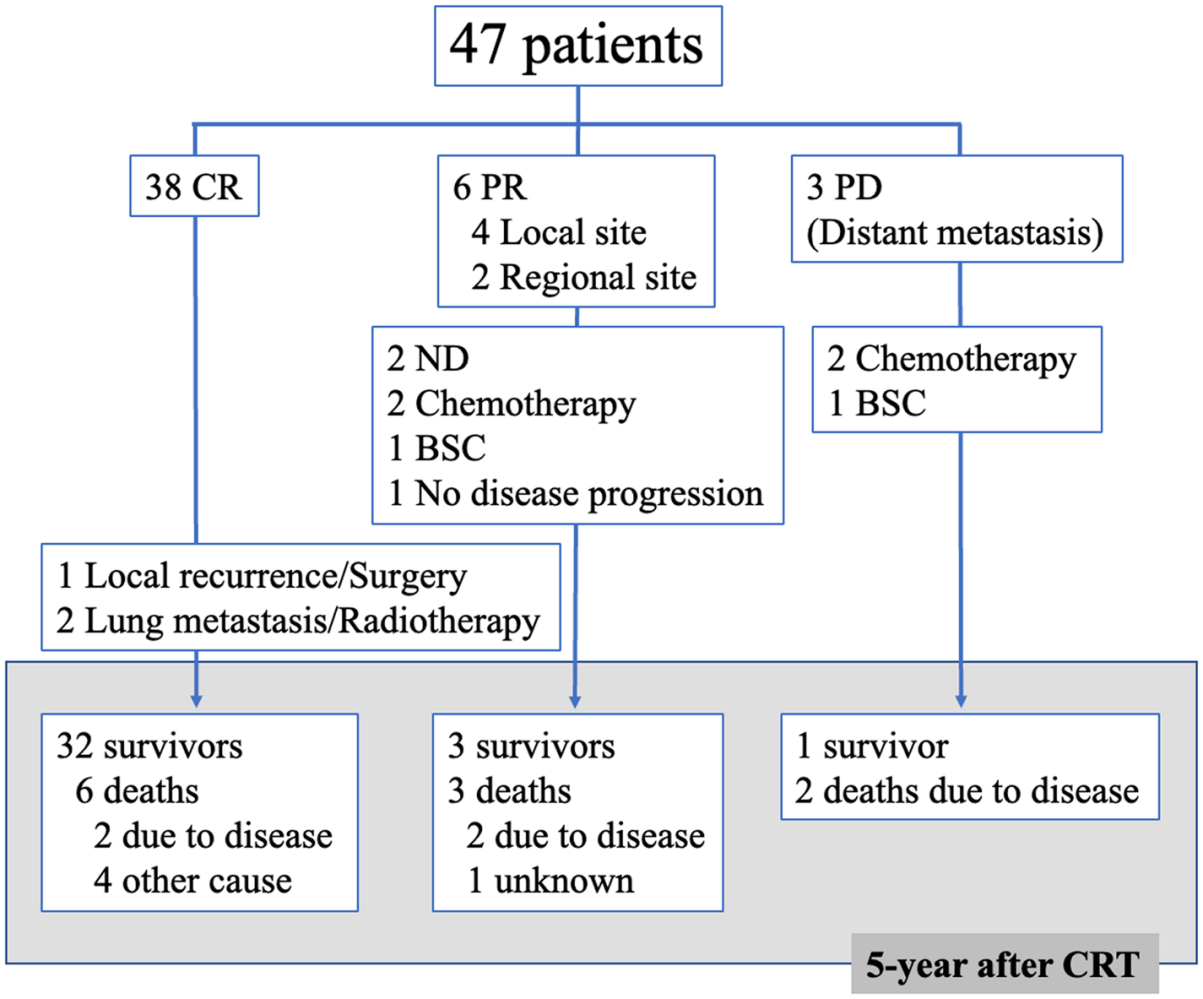
Flow diagram of 47 patients after concomitant chemoradiotherapy (CRT) CR, complete response; PR, partial response; PD, progressive disease; ND, neck dissection; BSC, best supportive care.

Figure 1 shows a flow diagram of the 47 patients after CCRT. Of the 38 patients, one underwent surgery after local recurrence and two received radiotherapy for lung metastases. Of the six patients who showed PR, two underwent neck dissection, two received chemotherapy, and one received the best supportive care. One patient showed no disease progression and was not treated. Of the three patients who showed PD, two received chemotherapy and one received the best supportive care. Finally, five years after CCRT, 36 patients survived, while six and five patients died due to the disease and other causes, respectively.

**Figure 2:**
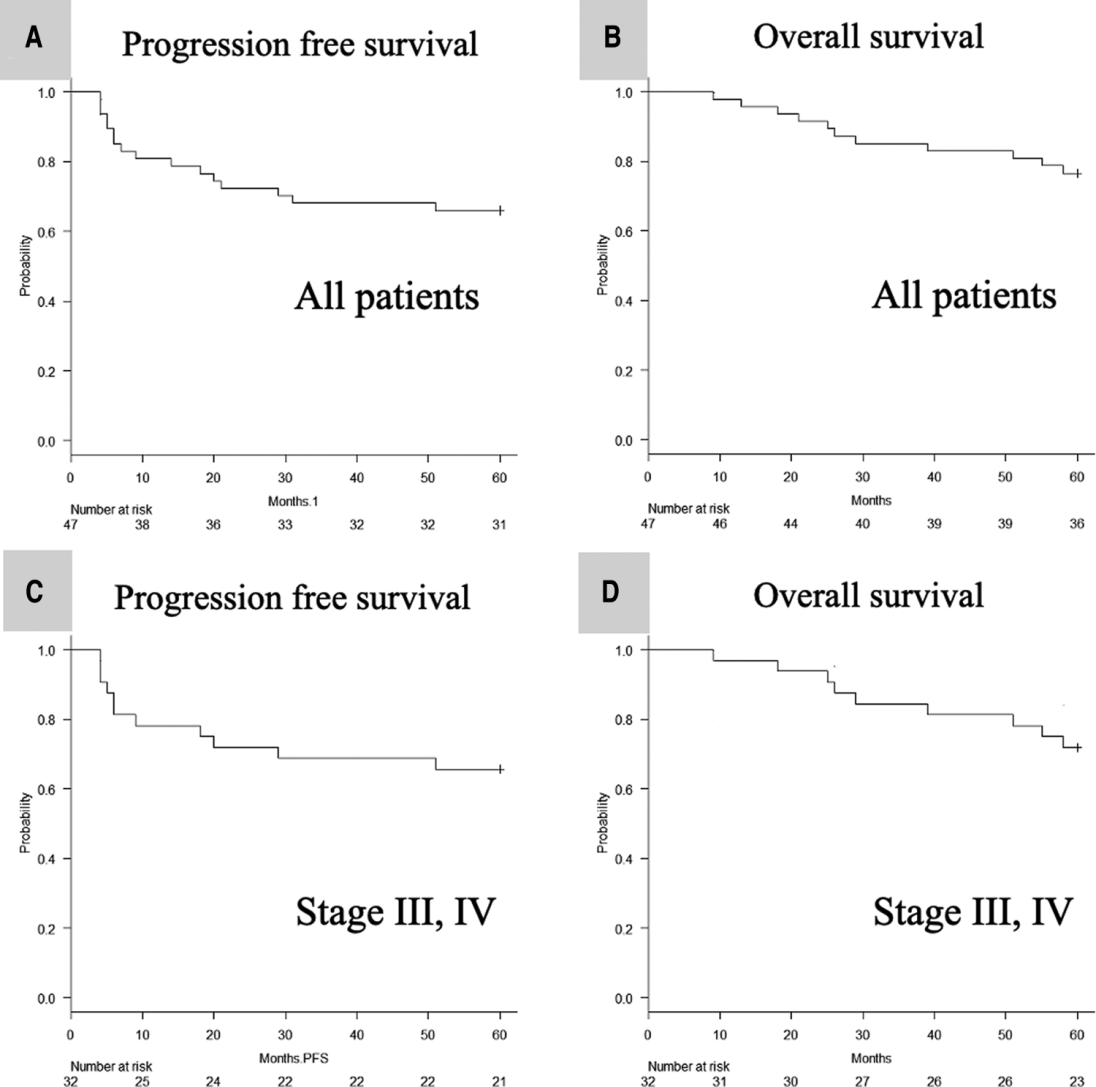
Kaplan–meier analysis of the 5-year PFS (**A**) and OS (**B**) of all 47 patients, and the 5-year PFS (**C**) and OS (**D**) of stage III, IV defined by UICC 8th patients. PFS, progression-free survival; OS, overall survival; UICC, Union for International Cancer Control.

The median follow-up period was 61 months (range: 9–73 months). The 5-year PFS (Figure 2A) and 5-year OS (Figure 2B) of all 47 patients were 66.0% (95% CI, 0.51 to 0.78) and 76.6% (95% CI, 0.62 to 0.86), respectively. The 5-year PFS (Figure 2C) and 5-year OS (Figure 2D) of patients with stage III/IV disease (UICC 8th version) were 65.6% (95% CI, 0.47–0.79) and 71.9% (95% CI, 0.53–0.84), respectively.

### Late adverse events

Thirty of the 47 patients (64%) showed no dysphagia (FOIS Level VI-VII), while 17 patients (36%) showed level V or less dysphagia (FOIS Level IV-V: nasopharynx, 2; oropharynx, 7; hypopharynx, 3; Level I-III: oropharynx, 1; hypopharynx, 3; larynx, 1) (data not shown). Multivariate analysis demonstrated that BMI < 22 and acute mucositis of grade 3 or 4 were significantly associated with long-term dysphagia of FOIS level V or less (Table 3).

**Table 3:** Univariate and multivariate analyses of dysphagia; FOIS level V or lower.

		Univariate		Multivariate	
Variable	No.	Hazard ratio (95% CI)	*P*	Hazard ratio (95% CI)	*P*
Age, ≥ 65 years	21	0.16 (0.35–3.84)	0.80	2.04 (0.46–9.10)	0.35
Sex, female	8	0.53 (0.10–2.99)	0.46	0.13 (0.01–1.32)	0.08
Body mass index, <22 kg/m^2^	23	2.75 (0.80–9.45)	0.11	5.81 (1.16–29.1)	0.03
Primary site, hypopharynx	17	0.94 (0.27–3.26)	0.93	1.48 (0.30–8.73)	0.63
T-category, T3 or T4	17	0.94 (0.27–3.26)	0.93	1.42 (0.23–8.73)	0.71
Stage classification, III or IV 8th	32	1.20 (0.33–4.36)	0.78	0.77 (0.11–5.51)	0.8
Acute mucositis, grade 3 or 4	25	3.14 (0.88–11.2)	0.08	8.07 (1.38–47.2)	0.02

Two of the 47 patients (4%) had dysphonia. Both were unable to speak (PSS-HN: never understandable) (data not shown): one patient had T2N1M0 hypopharyngeal cancer and underwent total laryngectomy due to local recurrence 9 months after CCRT while the other patient had T3N0M0 laryngeal cancer and underwent tracheostomy due to primary residue 6 months after CCRT. Nine (19%) and two (4%) of the 47 patients showed post-irradiation hypothyroidism and aspiration pneumonia, respectively.

## Discussion

### Survival outcomes of CCRT with a 3-weekly CDDP dose of 80 mg/m^2^

We retrospectively reviewed the patient data at 5 years after the previous phase 2 clinical trial of CCRT with a 3-weekly CDDP dose of 80 mg/m^2^ (4): the 5-year PFS and 5-year OS of all patients were 66% and 76%, respectively (Figures 2A, B). Moreover, even when limited to cases at an advanced stage (III, IV), the 5-year PFS and 5-year OS of all patients were as good as 66% and 72%, respectively (Figures 2C, D). The 5-year survival data of CCRT using a CDDP dose of 100 mg/m^2^ showed a PFS and OS of 28%–49.4% (6, 7) and 44%–76.8% (6–10), respectively. Although the CDDP dose in these studies was 100 mg/m^2^, the patient background characteristics differed across studies: Espeli et al. included patients who underwent postoperative CCRT (6); Nguyen-Tan et al. excluded patients with nasopharyngeal carcinoma (7); Han et al. included patients who underwent preoperative and postoperative CCRT and those with salivary gland carcinoma (8); Yang et al. included only nasopharyngeal carcinoma patients (9); and Forastiere et al. included only patients with laryngeal carcinoma (10). Therefore, we could not directly compare the current results with the findings of these reports. Nevertheless, the present results suggest that CCRT with a 3-weekly CDDP dose of 80 mg/m^2^ may be non-inferior to that using a CDDP dose of 100 mg/m^2^.

Other reports of CCRT with a 3-weekly CDDP dose of 80 mg/m^2^ have also shown somewhat favorable results, with 3-year PFS and OS of 55.9% (14) and 60%–80.8% (14, 15), respectively. Since the cumulative CDDP dose during RT was reported to be critical for survival outcome (16) and doses >200 mg/m^2^ were sufficient to achieve an additive effect with RT irrespective of the method of administration (17), the present PFS/OS data suggest that a reduction in the CDDP dose from 100 mg/m^2^ to 80 mg/m^2^ is reasonable. The current study did not investigate the nephro- and oto-toxicities possibly caused by CDDP. Since they are both cumulative and dose-dependent (18), dose reduction of CDDP would also reduce these sequelae which may sometimes interfere with post-CCRT chemotherapy. Thus, CCRT with a 3-weekly CDDP dose of 80 mg/m^2^ could be an alternative to a CDDP dose of 100 mg/m^2^. To confirm the non-inferiority of CDDP at a dose of 80 mg/m2 to 100 mg/m2, direct comparisons between these doses should be tested in future studies.

### Dysphagia among late adverse events

In the assessment of late adverse events, 17 of 47 (36%) patients rated dysphagia as FOIS level V or lower. While this percentage seems relatively higher than that in previous reports using 100 mg/m^2^ CDDP (5, 9), the absence of a quantitative definition of dysphagia in prior studies makes direct comparisons among studies difficult. However, since FOIS level V or lower dysphagia was associated with grade 3/4 acute mucositis (Table 3), these patients should receive preventive measures or care for acute mucositis.

In addition to acute mucositis, BMI < 22 was associated with late-stage dysphagia (Table 3). Overweight and increasing BMI predicted a lower risk of severe dysphagia after radiotherapy for head and neck cancer (19). Thus, swallowing training during/after CCRT should be considered for patients with a low BMI to prevent post-CCRT dysphagia.

## Conclusion

We retrospectively reviewed patient data 5 years after the previous phase 2 clinical trial of CCRT for HNSCC using a CDDP dose of 80 mg/m^2^. The 5-year PFS and 5-year OS were 66% and 76%, respectively, which may be comparable to the results obtained with a CDDP dose of 100 mg/m^2^. However, 36% of patients had dysphagia rated as FOIS V or lower even 5 years after CCRT, which was associated with acute mucositis during CCRT and low BMI. Caution and care for acute mucositis and swallowing training in patients with low BMI may be important for preventing late-stage dysphagia.

## Data Availability

The raw data supporting the conclusions of this article will be made available by the authors, without undue reservation.
